# Crystal structure and Hirshfeld-surface analysis of di­aqua­bis­(5-methyl-1*H*-1,2,4-triazole-3-carboxyl­ato)copper(II)

**DOI:** 10.1107/S2056989023010770

**Published:** 2024-01-01

**Authors:** Yuliia P. Petrenko, Oleksandr S. Vynohradov, Dmytro M. Khomenko, Roman O. Doroshchuk, Ilona V. Raspertova, Sergiu Shova, Rostyslav D. Lampeka

**Affiliations:** a Department of Chemistry, Taras Shevchenko National University of Kyiv, Volodymyrska str. 64/13, 01601 Kyiv, Ukraine; b Enamine Ltd., Chervonotkatska Street 78, Kyiv 02094, Ukraine; c "PetruPoni" Institute of Macromolecular Chemistry, Aleea Gr., GhicaVoda 41A, 700487 Iasi, Romania; University of Kentucky, USA

**Keywords:** copper, copper complexes, crystal structure, 1,2,4-triazole, X-ray crystallography, Hirshfeld surface analysis

## Abstract

The title compound, [Cu(H*L*)_2_(H_2_O)_2_] or [Cu(C_4_H_4_N_3_O_2_)_2_(H_2_O)_2_], is a mononuclear octa­hedral Cu^II^ complex based on 5-methyl-1*H*-1,2,4-triazole-3-carb­oxy­lic acid (H_2_ *L*). [Cu(H*L*)_2_(H_2_O)_2_] was synthesized by reaction of H_2_ *L* with copper(II) nitrate hexa­hydrate (2:1 stoichiometric ratio) in water under ambient conditions to produce clear light-blue crystals. The central Cu atom exhibits an N_2_O_4_ coordination environment in an elongated octa­hedral geometry provided by two bidentate H*L* ^−^ anions in the equatorial plane and two water mol­ecules in the axial positions. Hirshfeld surface analysis revealed that the most important contributions to the surface contacts are from H⋯O/O⋯H (33.1%), H⋯H (29.5%) and H⋯N/N⋯H (19.3%) inter­actions.

## Abstract 2

Herein, the synthesis and structure of the title compound, di­aqua­bis­(5-methyl-1*H*-1,2,4-triazole-3-carboxyl­ato)copper(II), which is a mononuclear complex based on 5-methyl-1*H*-1,2,4-triazole-3-carb­oxy­lic acid, are reported. A Hirshfeld surface analysis was also performed.

## 1. Chemical context

A few decades ago, 1,2,4-triazole-containing compounds became a focal point for both organic and inorganic chemists. It turned out that 1,2,4-triazoles are substances that show promising results as anti­bacterial, anti­cancer, anti­fungal, anti-inflammatory, and anti­viral agents and have miscellaneous biological activities (Opsomer & Dehaen, 2022 ▸; Strzelecka & Świątek, 2021 ▸; Karczmarzyk *et al.*, 2020 ▸). The presence of three nitro­gen atoms and the possibility of being involved in metal bonding, both in their acid and deprotonated forms, resulted in the synthesis and investigation of numerous coordination compounds based on 1,2,4-triazole derivatives (Haasnoot, 2000 ▸). As a result of the presence of the N–N bridging function in the triazole ring, these ligands can form polynuclear complexes with specific magnetic properties (Aromí *et al.*, 2011 ▸; Kitchen & Brooker, 2008 ▸; Klingele & Brooker, 2003 ▸; Petrenko *et al.*, 2020 ▸; 2021 ▸). Meanwhile, 1,2,4-triazole used as linker in ligands for MOF construction, is not usually involved in the formation of coordination bonds (Du *et al.*, 2005 ▸). The most widely used ligands of such type are 3-(2-pyrid­yl)-1,2,4-triazole derivatives, which readily form extremely stable planar coordination compounds with platinum (Chang *et al.*, 2006 ▸; Chen *et al.*, 2013 ▸) and palladium (Zakharchenko *et al.*, 2017 ▸; 2019 ▸; 2021 ▸), showing promising photoelectronic and catalytic properties, respectively. A carb­oxy­lic acid group connected directly to the 1,2,4-triazole ring could potentially play the same role as a 2-pyridyl moiety, forcing the formation of chelates. In addition, it should be noted that the presence of both carb­oxy­lic and 1,2,4-triazole groups as parts of one mol­ecule provides inter­esting theoret­ical insights into the structural peculiarities of these mol­ecules. This is mainly due to the possibility of 1,2,4-triazole existing in three tautomeric forms (Pagacz-Kostrzewa *et al.*, 2019 ▸, 2020 ▸). Generally, compounds containing a carb­oxy­lic function are probably the most important materials for high-throughput synthesis and 1,2,4-triazoles are not an exception. Recently, as part of our efforts to prepare new synthesis building blocks, we obtained a series of carb­oxy­lic acids and their derivatives (Khomenko *et al.*, 2022 ▸). One of those compounds was used to synthesize a copper complex. 
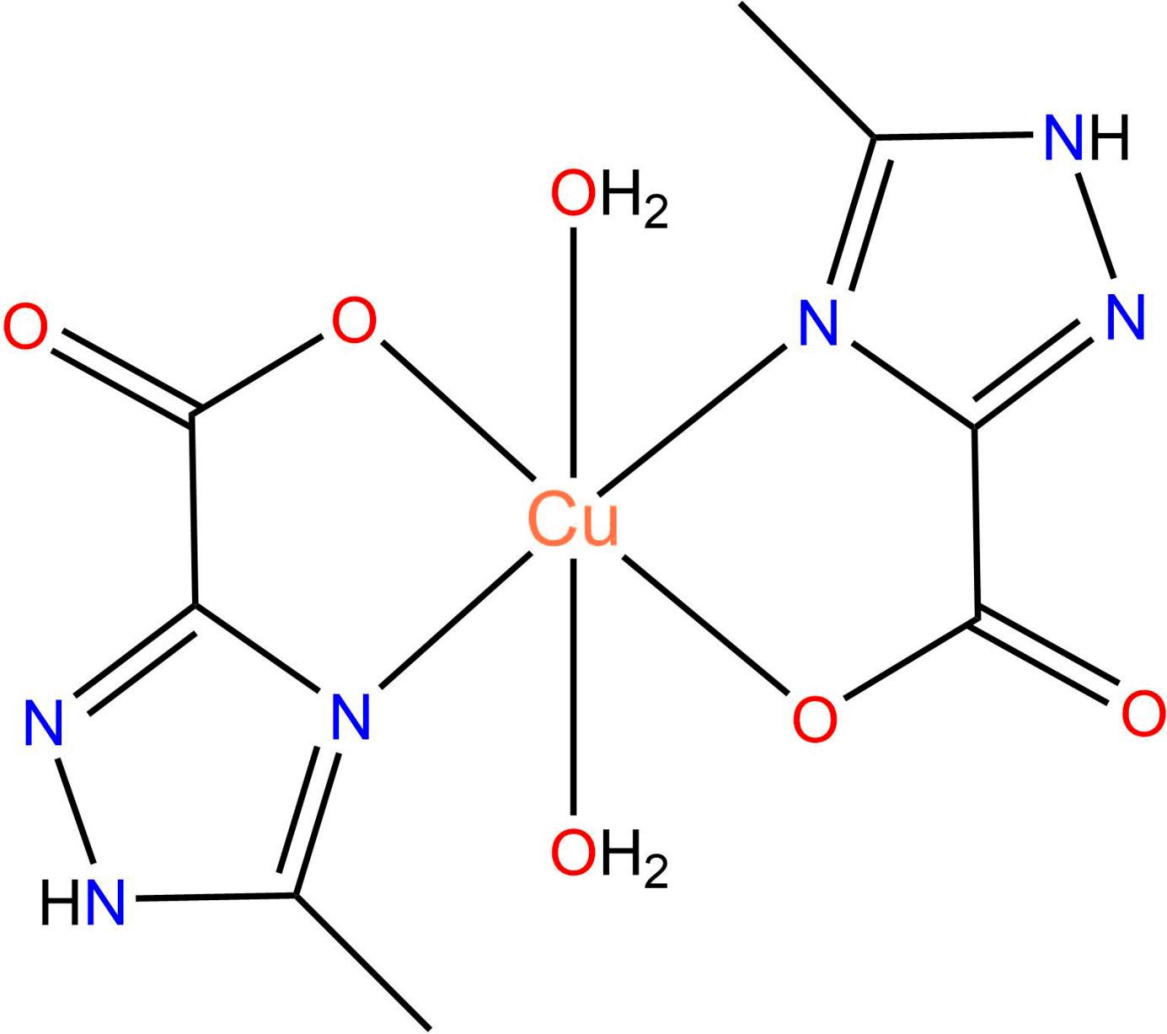


## 2. Structural commentary

**Figure 1:**
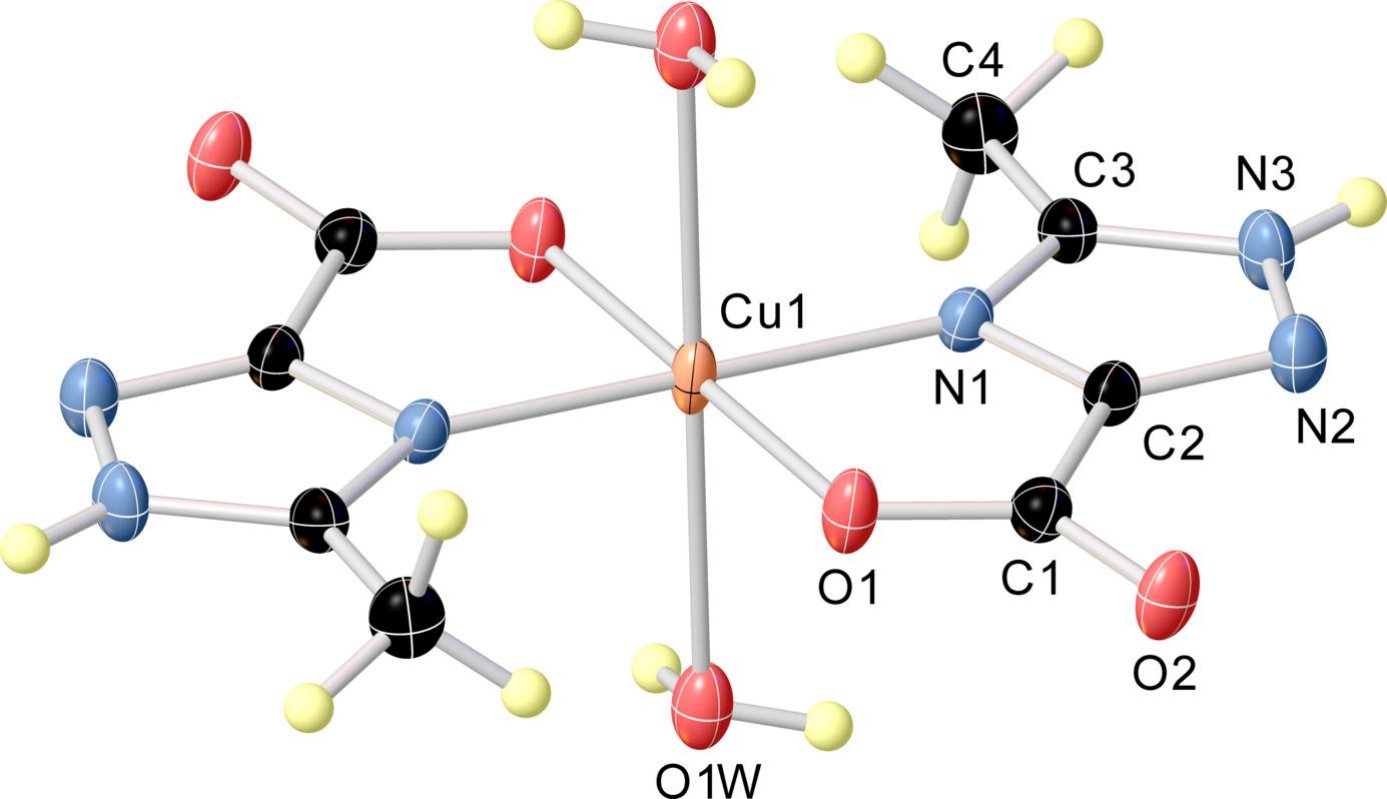
The mol­ecular structure of the title compound with the atom labelling. Displacement ellipsoids are drawn at the 50% probability level.

The mol­ecular structure of the title compound consists of a neutral complex unit [Cu(H*L*)_2_(H_2_O)_2_] (Fig. 1 ▸), where H*L* is the deprotonated 5-methyl-1*H*-1,2,4-triazole-3-carboxyl­ate ligand. The Cu^II^ atom occupies a special position (inversion centre), thus imposing crystallographic inversion symmetry on the complex. The central atom exhibits an N_2_O_4_ coordination environment in an elongated octa­hedral geometry provided by two bidentate L^−^ anions in the equatorial plane [Cu1—O1 = 1.9987 (12) Å, Cu1—N1 = 1.9603 (15) Å] and two water mol­ecules in the axial positions [Cu1—O1*W* = 2.5405 (15) Å]. It is worth noting that the structure of the title compound closely resembles those of earlier published analogous compounds with unsubstituted 1*H*-1,2,4-triazole-3-carboxyl­ate anions (Liu, 2007 ▸; Zhu *et al.*, 2007 ▸).

## 3. Supra­molecular features

**Figure 2:**
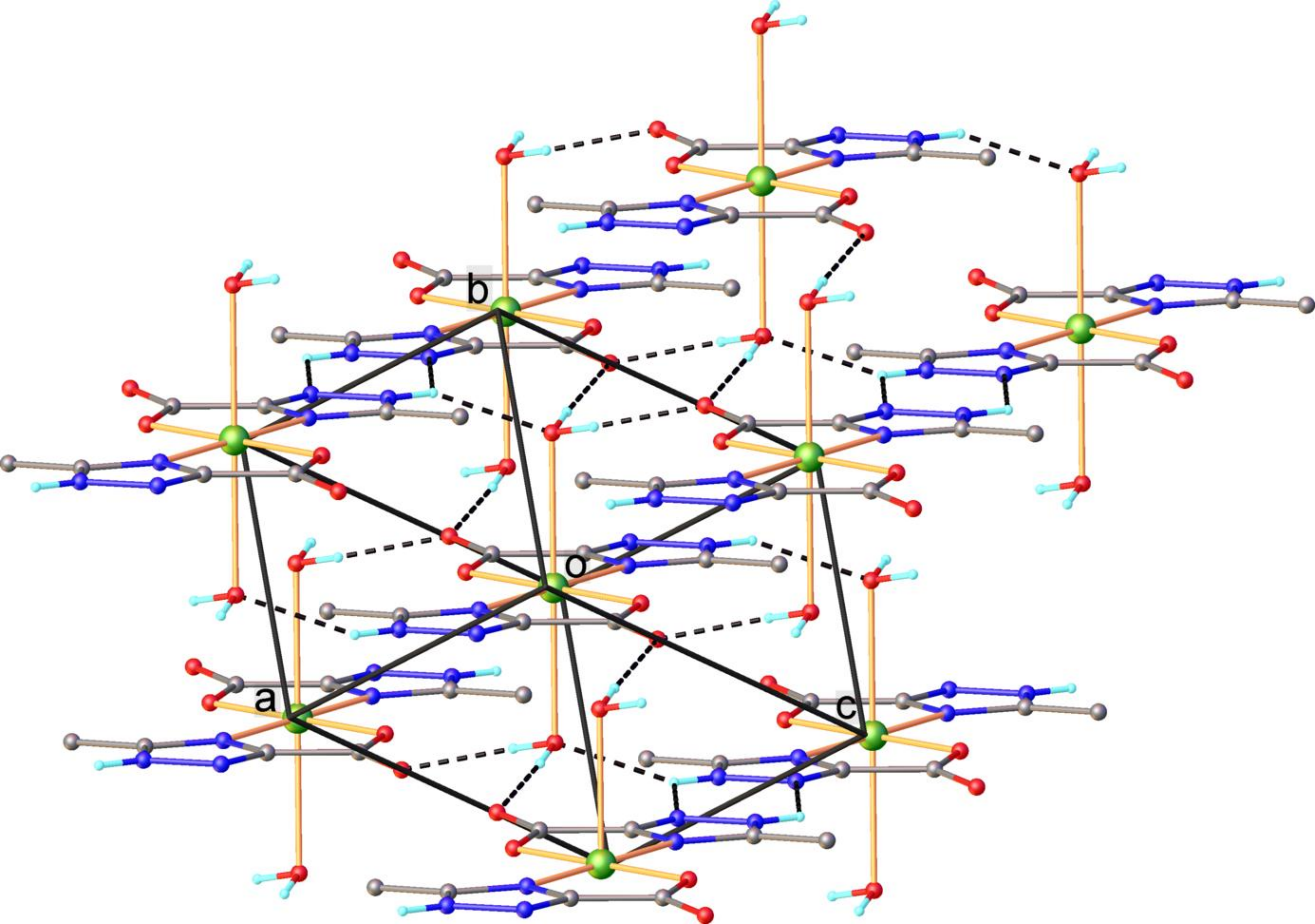
Partial view of the crystal packing showing the formation of the three-dimensional supra­molecular architecture.

In the crystal, the complex mol­ecules [Cu(H*L*)_2_(H_2_O)_2_] inter­act *via* numerous inter­molecular O—H⋯O and N—H⋯O hydrogen bonds (Table 1 ▸). Each NH group of the carboxyl­ate ligands is involved as the donor of a proton in bifurcated hydrogen bonding towards atom N2 and the coordinated water mol­ecule of the adjacent mol­ecule, while each O1*W* mol­ecule acts as donor in two hydrogen bonds with two O2 atoms of the symmetry-related complexes. Thus, all the potential hydrogen bonds are completely realized in the crystal, which results in the formation of a three-dimensional supra­molecular network, as shown in Fig. 2 ▸.

## 4. Hirshfeld surface analysis

**Figure 3:**
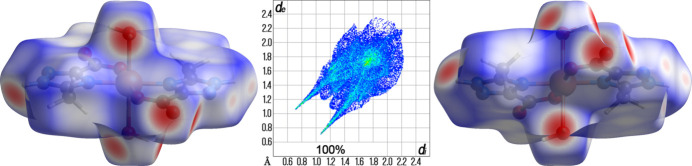
Two projections of the Hirshfeld surfaces mapped over *d*_norm_ showing the inter­molecular inter­actions within the mol­ecule and the full two-dimensional fingerprint plot for the title compound.

**Figure 4:**
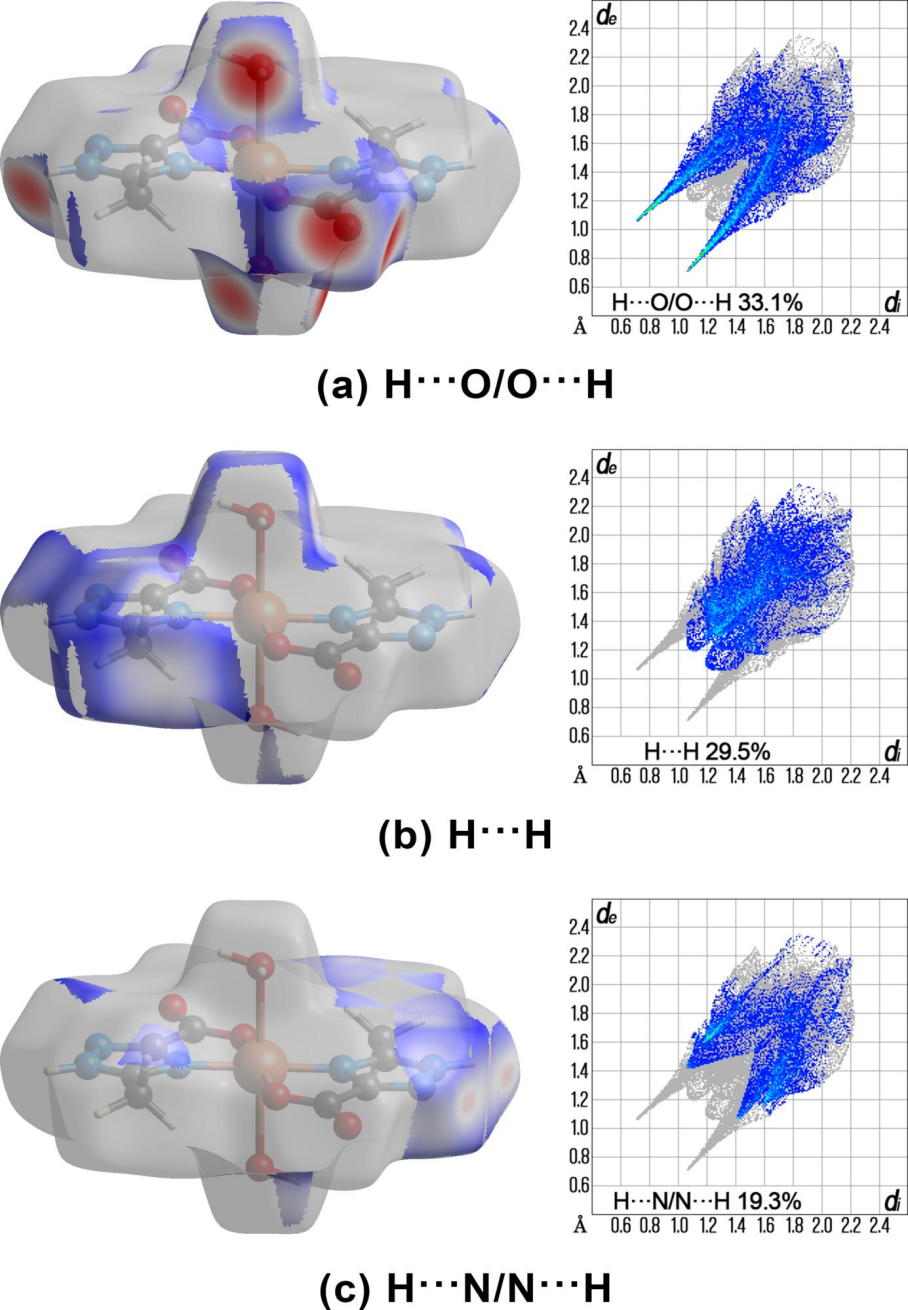
Hirshfeld surface representations with the function *d*_norm_ plotted onto the surface and the decomposed two-dimensional fingerprint plots for selected inter­actions.

A Hirshfeld surface analysis was performed and the associated two-dimensional fingerprint plots were generated using *CrystalExplorer* 17.5 software (Spackman *et al.*, 2021 ▸), with a standard resolution of the three-dimensional *d*_norm_ surfaces. There are 20 red spots on the *d*_norm_ surface (Fig. 3 ▸). The dark-red spots arise as a result of short inter­atomic contacts and represent contacts shorter than the sum of van der Waals radii, while the other weaker inter­molecular inter­actions appear as light-red spots. The Hirshfeld surfaces mapped over *d*_norm_ are shown for the H⋯O/O⋯H, H⋯H and H⋯N/N⋯H contacts, and the decomposed two-dimensional fingerprint plots of different types of inter­actions are given in Fig. 4 ▸. All short inter­atomic contacts are in the range of 1.797–2.505 Å. The shortest contacts are OH⋯O and the longest contacts are NH⋯N. The most abundant contributions to the overall crystal packing are from H⋯O/O⋯H (33.1%), H⋯H (29.5%) and H⋯N/N⋯H (19.3%). There is a small contribution by other weak inter­molecular contacts: H⋯C/C⋯H (4.6%), O⋯N/N⋯O (4.2%), O⋯C/C⋯O (3.3%), C⋯C (2.2%), O⋯O (1.8%), N⋯C/C⋯N (1.4%) and N⋯N (0.5%). In addition, qu­anti­tative physical properties of the Hirshfeld surface for this compound were obtained, such as mol­ecular volume (303.40 Å^3^), surface area (289.05 Å^2^), globularity (0.755), as well as asphericity (0.087).

## 5. Database survey

A search of the Cambridge Structural Database (CSD version 5.43, November 2021; Groom *et al.*, 2016 ▸) for the moiety including a transition metal coordinated by the N and O atoms of the 1*H*-1,2,4-triazole-3-carboxyl­ate anion in a bidentate way revealed 22 hits. Most similar to the title compound are mononuclear complexes with two unsubstituted 1*H*-1,2,4-triazole-3-carboxyl­ate anions and two water mol­ecules in axial positions: Mn^II^ [GEVKAW (Yan *et al.*, 2018 ▸)]; Zn^II^ [RIRVIY (Liu, 2007 ▸)]; Cd^II^ [XIRZOO (Zhu *et al.*, 2008 ▸)]; Cu^II^ [YIQROG (Zhu *et al.*, 2007 ▸) and YIQROG01 (Liu, 2007 ▸)]. Other compounds with a close relation to the title complex are mononuclear complexes with 5-substituted 1*H*-1,2,4-triazole-3-carboxyl­ate anions. In all cases, the substituent was the NH_2_ group: Mn^II^ [HEDWIZ (Yang *et al.*, 2019 ▸)], Mn^II^ dihydrate [OPOMAJ (Liu *et al.*, 2015 ▸)], Cd^II^ [ISACEL (Wang *et al.*, 2011 ▸)], Co^II^ dihydrate [ONILIJ (Li *et al.*, 2021 ▸)], Zn^II^ based on 5-amino-1*H*-1,2,4-triazole-3-carboxyl­ate anion and with only one coordinated water mol­ecule [OPOLUC (Liu *et al.*, 2015 ▸)].

## 6. Synthesis and crystallization

**H_2_** ***L:*** LiH*L* (Khomenko *et al.*, 2022 ▸) (1.33 g, 10 mmol) was dissolved in H_2_O (10 ml). The obtained solution was cooled and slowly acidified with concentrated HCl (1 ml), maintaining the temperature between 273 and 278 K. The precipitation of colourless crystals occurred after addition of all the HCl. The reaction mixture was additionally stirred for 15 min at low temperature. Then, the precipitate was filtered off, washed with cold water and dried *in vacuo*. Yield 0.76 g (60%). ^1^H NMR (400 MHz, D_2_O): δ 2.61 (*s*, 3H) ppm. IR data (in KBr, cm^−1^): 3330, 1648, 1567, 1509, 1418, 1313, 1103, 835. Elemental analysis: analysis calculated for C_4_H_5_N_3_O_2_ (127.10): C, 37.80%; H, 3.97%; N, 33.06%. Found: C, 37.41%; H, 3.65%; N, 32.71%.

**[Cu(H** ***L*** **)_2_(H_2_O)_2_]:** A solution of Cu(NO_3_)_2_ **·**6H_2_O (0.148 g, 0.5 mmol) in H_2_O (5 ml) was added to an aqueous solution of H_2_ *L* (0.127 g, 13 ml, 1 mmol) to give a clear blue solution. The blue crystals obtained after 2 days were filtered off, washed with water and dried in air. Yield 0.140 g (80%). IR data (in KBr, cm^−1^): 3330, 1648, 1557, 1509, 1418, 1304, 1113, 835. Elemental analysis: analysis calculated for C_8_H_12_CuN_6_O_6_ (351.77): C, 27.32%; H, 3.44%; N, 23.89%. Found: C, 27.30%; H, 3.45%; N, 23.82%.

IR and ^1^H NMR spectra of 5-methyl-1*H*-1,2,4-triazole-3-carb­oxy­lic acid are given in the supporting information for this article.

## 7. Refinement

Crystal data, data collection and structure refinement details are summarized in Table 2 ▸. H atoms were found in difference-Fourier maps, but subsequently included in the refinement using riding models, with constrained distances set to 0.96 Å (*R*CH_3_), 0.86 Å (N*sp* ^2^—H), and 0.85 (OH_2_). *U_i_*_so_(H) parameters were set to values of either 1.2*U*_eq_ or 1.5*U*_eq_ (*R*CH_3_, OH_2_) of the attached atom.

## Supplementary Material

Crystal structure: contains datablock(s) I. DOI: 10.1107/S2056989023010770/pk2702sup1.cif

Structure factors: contains datablock(s) I. DOI: 10.1107/S2056989023010770/pk2702Isup2.hkl

Supporting information file. DOI: 10.1107/S2056989023010770/pk2702Isup3.cdx

IR spectrum of 5-methyl-1H-1,2,4-triazole-3-carboxylic acid. DOI: 10.1107/S2056989023010770/pk2702sup4.jpg

1H NMR spectrum of 5-methyl-1H-1,2,4-triazole-3-carboxylic acid. DOI: 10.1107/S2056989023010770/pk2702sup5.tif

CCDC reference: 2314480

Additional supporting information:  crystallographic information; 3D view; checkCIF report

## Figures and Tables

**Table 1:** Hydrogen-bond geometry (Å, °)

*D*—H⋯*A*	*D*—H	H⋯*A*	*D*⋯*A*	*D*—H⋯*A*
N3—H3⋯O1*W* ^i^	0.86	2.06	2.822 (2)	147
N3—H3⋯N2^ii^	0.86	2.58	3.121 (2)	122
O1*W*—H1*WB*⋯O2^iii^	0.85	1.93	2.781 (2)	175
O1*W*—H1*WA*⋯O2^iv^	0.85	1.92	2.737 (2)	160

Symmetry codes: (i) 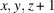 ; (ii) 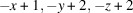 ; (iii) 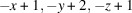 ; (iv) 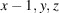 .

**Table 2:** Experimental details

Crystal data	
Chemical formula	[Cu(C_4_H_4_N_3_O_2_)_2_(H_2_O)_2_]
*M*_r_	351.78
Crystal system, space group	Triclinic, *P* 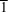
Temperature (K)	200
*a*, *b*, *c* (Å)	6.8465 (4), 7.1097 (7), 7.2090 (5)
α, β, γ (°)	79.267 (7), 83.193 (6), 64.076 (8)
*V* (Å^3^)	309.80 (5)
*Z*	1
Radiation type	Mo *K*α
μ (mm^−1^)	1.81
Crystal size (mm)	0.45 × 0.1 × 0.1

Data collection	
Diffractometer	Xcalibur, Eos
Absorption correction	Multi-scan (*CrysAlis PRO*; Agilent, 2012 ▸)
*T*_min_, *T*_max_	0.774, 1.000
No. of measured, independent and observed [*I* > 2σ(*I*)] reflections	2216, 1397, 1381
*R*_int_	0.016
(sin θ/λ)_max_ (Å^−1^)	0.681

Refinement	
*R*[*F* ^2^ > 2σ(*F* ^2^)], *wR*(*F* ^2^), *S*	0.024, 0.062, 1.11
No. of reflections	1397
No. of parameters	101
H-atom treatment	H-atom parameters constrained
Δρ_max_, Δρ_min_ (e Å^−3^)	0.35, −0.38

Computer programs: *CrysAlis PRO* (Agilent, 2012 ▸), *SHELXT2018/2* (Sheldrick, 2015*a* ▸), *SHELXL2018/3* (Sheldrick, 2015*b* ▸) and *OLEX2* (Dolomanov *et al.*, 2009 ▸).

## supplementary crystallographic information

### Crystal data

**Table:**

[Cu(C_4_H_4_N_3_O_2_)_2_(H_2_O)_2_]	*Z* = 1
*M**_r_* = 351.78	*F*(000) = 179
Triclinic, *P*1	*D*_x_ = 1.886 Mg m^−^^3^
*a* = 6.8465 (4) Å	Mo *K*α radiation, λ = 0.71073 Å
*b* = 7.1097 (7) Å	Cell parameters from 1444 reflections
*c* = 7.2090 (5) Å	θ = 2.9–28.9°
α = 79.267 (7)°	µ = 1.81 mm^−^^1^
β = 83.193 (6)°	*T* = 200 K
γ = 64.076 (8)°	Prism, clear light blue
*V* = 309.80 (5) Å^3^	0.45 × 0.1 × 0.1 mm

### Data collection

**Table:**

Xcalibur, Eos diffractometer	1397 independent reflections
Radiation source: Enhance (Mo) X-ray Source	1381 reflections with *I* > 2σ(*I*)
Graphite monochromator	*R*_int_ = 0.016
Detector resolution: 16.1593 pixels mm^-1^	θ_max_ = 29.0°, θ_min_ = 2.9°
ω scans	*h* = −8→9
Absorption correction: multi-scan (*CrysAlisPro*; Agilent, 2012)	*k* = −9→9
*T*_min_ = 0.774, *T*_max_ = 1.000	*l* = −9→9
2216 measured reflections	

### Refinement

**Table:**

Refinement on *F*^2^	Primary atom site location: dual
Least-squares matrix: full	Hydrogen site location: mixed
*R*[*F*^2^ > 2σ(*F*^2^)] = 0.024	H-atom parameters constrained
*wR*(*F*^2^) = 0.062	*w* = 1/[σ^2^(*F*_o_^2^) + (0.0239*P*)^2^ + 0.1883*P*] where *P* = (*F*_o_^2^ + 2*F*_c_^2^)/3
*S* = 1.11	(Δ/σ)_max_ < 0.001
1397 reflections	Δρ_max_ = 0.35 e Å^−^^3^
101 parameters	Δρ_min_ = −0.38 e Å^−^^3^
0 restraints	

### Special details

**Table:**

Geometry. All esds (except the esd in the dihedral angle between two l.s. planes) are estimated using the full covariance matrix. The cell esds are taken into account individually in the estimation of esds in distances, angles and torsion angles; correlations between esds in cell parameters are only used when they are defined by crystal symmetry. An approximate (isotropic) treatment of cell esds is used for estimating esds involving l.s. planes.

### Fractional atomic coordinates and isotropic or equivalent isotropic displacement parameters (Å^2^)

**Table:**

	*x*	*y*	*z*	*U*_iso_*/*U*_eq_	
Cu1	0.500000	0.500000	0.500000	0.01665 (11)	
O1	0.7195 (2)	0.6171 (2)	0.41929 (19)	0.0200 (3)	
O1W	0.2171 (2)	0.8296 (2)	0.31785 (19)	0.0211 (3)	
H1WA	0.100833	0.815339	0.353434	0.032*	
H1WB	0.208435	0.929486	0.372183	0.032*	
O2	0.8357 (2)	0.8422 (2)	0.49419 (19)	0.0216 (3)	
N1	0.4392 (2)	0.6629 (2)	0.7081 (2)	0.0148 (3)	
N2	0.5479 (2)	0.8664 (2)	0.8331 (2)	0.0184 (3)	
N3	0.3859 (2)	0.8329 (2)	0.9412 (2)	0.0187 (3)	
H3	0.332405	0.882766	1.045013	0.022*	
C1	0.7246 (3)	0.7403 (3)	0.5225 (3)	0.0161 (3)	
C2	0.5727 (3)	0.7621 (3)	0.6937 (2)	0.0151 (3)	
C3	0.3199 (3)	0.7127 (3)	0.8661 (2)	0.0160 (3)	
C4	0.1414 (3)	0.6522 (3)	0.9429 (3)	0.0223 (4)	
H4A	0.011749	0.743962	0.876397	0.033*	
H4B	0.115402	0.664910	1.074796	0.033*	
H4C	0.181446	0.508565	0.927290	0.033*	

### Atomic displacement parameters (Å^2^)

**Table:**

	*U*^11^	*U*^22^	*U*^33^	*U*^12^	*U*^13^	*U*^23^
Cu1	0.02239 (18)	0.01979 (18)	0.01580 (17)	−0.01517 (14)	0.00516 (11)	−0.00932 (12)
O1	0.0244 (7)	0.0226 (7)	0.0205 (7)	−0.0158 (6)	0.0067 (5)	−0.0108 (5)
O1W	0.0233 (7)	0.0253 (7)	0.0215 (7)	−0.0154 (6)	0.0071 (5)	−0.0113 (6)
O2	0.0219 (7)	0.0232 (7)	0.0275 (7)	−0.0161 (6)	0.0066 (5)	−0.0102 (6)
N1	0.0170 (7)	0.0143 (7)	0.0156 (7)	−0.0087 (6)	0.0009 (5)	−0.0038 (6)
N2	0.0198 (7)	0.0214 (8)	0.0188 (8)	−0.0121 (7)	0.0022 (6)	−0.0076 (6)
N3	0.0215 (8)	0.0221 (8)	0.0164 (8)	−0.0114 (7)	0.0041 (6)	−0.0098 (6)
C1	0.0168 (8)	0.0150 (8)	0.0165 (8)	−0.0067 (7)	−0.0001 (6)	−0.0034 (7)
C2	0.0162 (8)	0.0148 (8)	0.0166 (8)	−0.0085 (7)	−0.0006 (6)	−0.0033 (7)
C3	0.0180 (8)	0.0151 (8)	0.0147 (8)	−0.0063 (7)	−0.0002 (6)	−0.0041 (7)
C4	0.0216 (9)	0.0270 (10)	0.0216 (9)	−0.0140 (8)	0.0041 (7)	−0.0056 (8)

### Geometric parameters (Å, º)

**Table:**

Cu1—O1	1.9987 (12)	N1—C2	1.363 (2)
Cu1—O1^i^	1.9987 (12)	N1—C3	1.329 (2)
Cu1—O1W	2.5405 (15)	N2—N3	1.361 (2)
Cu1—N1	1.9603 (15)	N2—C2	1.310 (2)
Cu1—N1^i^	1.9603 (15)	N3—C3	1.340 (2)
O1—C1	1.264 (2)	C1—C2	1.501 (2)
O2—C1	1.239 (2)	C3—C4	1.481 (2)
			
O1^i^—Cu1—O1	180.0	C3—N1—C2	104.36 (14)
O1—Cu1—O1W	89.09 (5)	C2—N2—N3	101.94 (14)
O1^i^—Cu1—O1W	90.91 (5)	C3—N3—N2	111.67 (15)
N1^i^—Cu1—O1	97.00 (5)	O1—C1—C2	113.53 (15)
N1^i^—Cu1—O1^i^	83.00 (5)	O2—C1—O1	127.10 (17)
N1—Cu1—O1^i^	97.00 (5)	O2—C1—C2	119.35 (16)
N1—Cu1—O1	83.00 (5)	N1—C2—C1	116.79 (15)
N1^i^—Cu1—O1W	92.71 (5)	N2—C2—N1	114.35 (16)
N1—Cu1—O1W	87.29 (5)	N2—C2—C1	128.86 (16)
N1^i^—Cu1—N1	180.0	N1—C3—N3	107.67 (15)
C1—O1—Cu1	115.49 (11)	N1—C3—C4	126.36 (16)
C2—N1—Cu1	111.00 (11)	N3—C3—C4	125.95 (16)
C3—N1—Cu1	144.60 (12)		

Symmetry code: (i) −*x*+1, −*y*+1, −*z*+1.

### Hydrogen-bond geometry (Å, º)

**Table:**

*D*—H···*A*	*D*—H	H···*A*	*D*···*A*	*D*—H···*A*
N3—H3···O1*W*^ii^	0.86	2.06	2.822 (2)	147
N3—H3···N2^iii^	0.86	2.58	3.121 (2)	122
O1*W*—H1*WB*···O2^iv^	0.85	1.93	2.781 (2)	175
O1*W*—H1*WA*···O2^v^	0.85	1.92	2.737 (2)	160
C4—H4*B*···O1*W*^ii^	0.96	2.59	3.367 (2)	139

Symmetry codes: (ii) *x*, *y*, *z*+1; (iii) −*x*+1, −*y*+2, −*z*+2; (iv) −*x*+1, −*y*+2, −*z*+1; (v) *x*−1, *y*, *z*.
