# Supplementary figures and images for: Crystal structure and Hirshfeld-surface analysis of di­aqua­bis­(5-methyl-1H-1,2,4-triazole-3-carboxyl­ato)copper(II)
Source: Acta Crystallogr E Crystallogr Commun. 2024 Jan 1;80(Pt 1):54–7. doi: 10.1107/S2056989023010770 (PMC10833364; doi:10.1107/S2056989023010770)

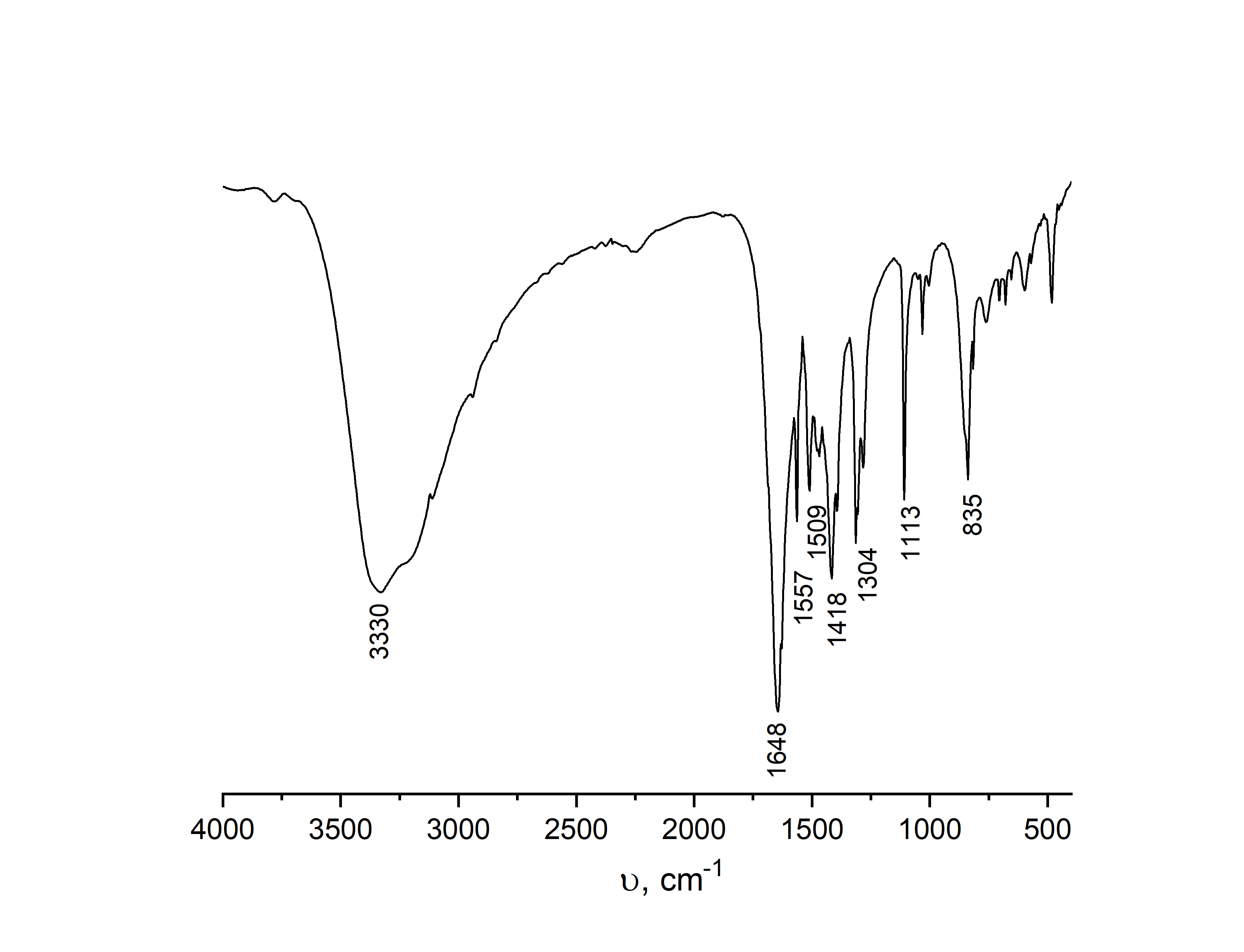

Supplement: Supplementary file 4 [file e-80-00054-sup4.jpg]

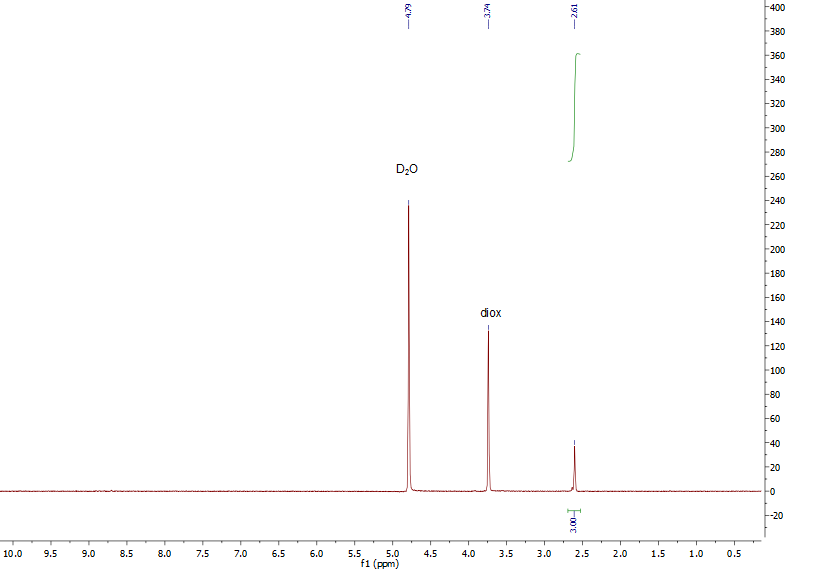

Supplement: Supplementary file 5 [file e-80-00054-sup5.tif]
